# Preschoolers’ Win–Stay/Lose–Shift Strategy Use in the Children’s Gambling Task

**DOI:** 10.3390/bs16010023

**Published:** 2025-12-22

**Authors:** Seokyung Kim, Stephanie M. Carlson

**Affiliations:** Institute of Child Development, University of Minnesota—Twin Cities, Minneapolis, MN 55455, USA

**Keywords:** Children’s Gambling Task, win–stay/lose–shift strategies, executive function, metacognition, cognitive self-regulation, adaptive decision-making

## Abstract

Adaptive decision-making requires flexible responses to environmental feedback and integration of information over time. Win–stay/lose–shift strategies describe immediate responses to outcomes: repeating a choice after a win (win–stay) or switching after a loss (lose–shift). Although these strategies have been examined using the Preschool Gambling Task, no study has investigated them in the widely used Children’s Gambling Task (CGT) to our knowledge. Our primary aim was to examine whether preschoolers adjust these strategies as they learn environmental contingencies. Using a shortened (40-trial) CGT with one advantageous deck (smaller rewards, smaller losses, net gains) and one disadvantageous deck (bigger rewards, bigger losses, net losses), we investigated strategy use in typically developing 3–5-year-old children (*N* = 98; 63% female; 88% white; 96% college-educated caregivers). A secondary aim examined whether higher cognitive self-regulation—executive function (EF) and metacognition—improves children’s effective deck-specific strategy use. Results showed preschoolers increasingly adopted win–stay in the advantageous deck but showed reduced lose–shift over time regardless of deck. Three-year-olds used significantly less lose–shift than 4-to-5-year-olds. Critically, metacognition—but not EF—uniquely predicted deck-specific strategies: children who knew which deck was better used more win–stay in the advantageous deck and more lose–shift in the disadvantageous deck, controlling for age, verbal ability, and strategy use in the opposite deck. These findings illuminate preschoolers’ strategic adaptation and highlight metacognition as a key driver of adaptive decision-making.

## 1. Introduction

A fundamental challenge for young children is to gradually become competent at recognizing patterns in their environment and modifying their behavior based on environmental feedback (Defoe et al., 2015; Nussenbaum & Hartley, 2019). It initially starts with responding to immediate outcomes, but gradually integrates information over time to ultimately identify and choose options that reliably lead to preferred outcomes and avoid options that lead to unfavorable outcomes (Daw et al., 2006; Sutton & Barto, 2018). Such adaptive decision-making skills are associated with multiple positive outcomes in adulthood, such as higher self-control, better social-cognitive and emotional coping abilities, and protection against various health vulnerabilities, including high BMI (Casey et al., 2011). Therefore, it is imperative to understand how adaptive decision-making develops in early childhood and lays the foundation for lifelong learning and problem-solving skills. In the current study, we used the Children’s Gambling Task (Kerr & Zelazo, 2004) to examine the mechanisms underlying preschoolers’ learning from feedback in an uncertain decision-making context with reward and loss contingencies.

### 1.1. Children’s Gambling Task

One of the most widely used tasks that mimics real-life complexities and uncertainties is the Iowa Gambling Task (IGT; Bechara et al., 1994). The IGT was originally created to test risk-taking behavior in neurological patients compared to healthy controls in an emotionally charged environment. Participants were given four decks of cards and told to choose one card per trial, without knowing the total number of trials (100 trials). Each deck operated with different rules for gains and losses of game money, varying in the size of immediate gains, the size and frequency of delayed losses, and the net outcome. A challenge Bechara et al. (1994) introduced in this task was that two disadvantageous decks appeared more appealing because they offered higher immediate rewards than the two advantageous decks. However, repeatedly choosing the two disadvantageous decks resulted in net losses because their unpredictable losses were larger on average than those from the advantageous decks. This setup creates a conflict between short-term gains versus long-term outcomes, requiring individuals to learn the game structure through accumulated feedback rather than reacting to single outcomes.

Subsequently, the IGT has been adapted for developmental populations, including a simplified version for IGT (Cassotti et al., 2011), the Soochow Gambling Task (Aïte et al., 2012) and the Hungry Donkey Task (first introduced by Crone & van der Molen, 2004) for school-age children as well as the Children’s Gambling Task (CGT; Kerr & Zelazo, 2004)—the main task used in our study—and the Preschool Gambling Task (PGT; first introduced by Garon & Moore, 2004) for young children. The CGT simplified the original IGT by reducing the number of trials by half (to 50 trials), limiting the number of decks to two while maintaining consistent loss frequencies across both decks, and using concrete, child-friendly rewards (e.g., M&Ms) instead of game money. As noted, all of these variants differ from another well-known decision-making paradigm—delay of gratification (Mischel, 1974; Thompson et al., 1997; Prencipe & Zelazo, 2005)—in terms of task complexity. Whereas IGT variants require individuals to learn the underlying reward structure through experience, the delay of gratification task is more straightforward, as simply choosing to postpone an immediate reward brings a larger reward later.

Earlier works on CGT have shown a coherent increase in advantageous deck choices across development. In Kerr and Zelazo’s (2004) study, which included five 10-trial blocks, 4-year-olds made more advantageous deck choices than 3-year-olds on later blocks. Specifically, 4-year-olds made significantly more advantageous choices than would be expected by chance on Blocks 3 and 5, whereas 3-year-olds made significantly fewer advantageous choices than would be expected by chance on Blocks 3 and 4. This age effect was reliable, supported by subsequent studies using the CGT (Andrews & Moussaumai, 2015; Bunch et al., 2007; Delgado et al., 2022; Gao et al., 2009; Garon & Doucet, 2024; Heilman et al., 2008; Hongwanishkul et al., 2005; Mata et al., 2013a) and non-CGT preschool variants (Bunch & Andrews, 2012; Garon & English, 2022; Garon et al., 2023; Garon & Moore, 2007).

In contrast to the robust age effect, evidence on gender differences in CGT performance has been more variable. In Kerr and Zelazo’s (2004) study, a marginal male advantage was reported among 3-year-olds. This finding was consistent with previous findings of male outperformance on the reversal learning task in younger preschoolers (Overman, 2004), which was replicated in a few other studies using the CGT (Heilman et al., 2008; Gao et al., 2009). However, the majority of research using either CGT or a non-CGT preschool variant has reported no gender difference (Andrews & Moussaumai, 2015; Delgado et al., 2022; Garon & English, 2022; Garon et al., 2023; Hongwanishkul et al., 2005; Mata et al., 2013a), and some studies have even shown a female advantage (e.g., Bunch et al., 2007; Garon & Moore, 2004). Therefore, the specific role of gender in CGT performance remains inconclusive.

### 1.2. Win–Stay and Lose–Shift as a Decision-Making Strategy

Much of this research on children’s decision-making in child-friendly IGT variants has focused on overall deck preferences—such as the difference between advantageous and disadvantageous deck selections—rather than on the specific strategies children deploy. This limitation has prompted researchers to investigate children’s decision-making processes through explore–exploit and win–stay/lose–shift indices, customizing their operationalization and computation to fit the unique features of each IGT variant.

Regarding the first strategy, exploration involves trying out various options and is typically used when knowledge is limited and uncertainty is high, whereas exploitation involves repeatedly choosing the option that provides the greatest reward, typically under conditions of high knowledge and low uncertainty (Daw et al., 2006; Kim & Carlson, 2024, for review). Adults generally start by exploring to learn about the environment and then gradually shift toward exploitation as they gather more information, balancing both processes. Developmental studies have shown that young children engage in broad and intensive exploration—often at the expense of exploitation—and that this exploratory tendency declines with age (Gopnik, 2020). However, more recent findings suggest that children also begin to develop exploitation in CGT-like tasks (see Section 1.1) as well as in other persistence-related choice tasks (Kim et al., 2025). In CGT contexts, exploration can be operationalized as switching decks between two consecutive trials, while exploitation can be operationalized as choosing the same deck on two consecutive trials (Garon & English, 2022).

As for the second strategy, which is the main interest of our study, win–stay refers to repeating the same choice after a win, and lose–shift refers to changing to a new choice after a loss. This is a very common adult decision-making strategy in uncertain situations (Paulus et al., 2001). An increase in win–stay and a decrease in lose–shift responses typically indicate that participants are learning the task structure (Wurm et al., 2022). As participants gain a better internal representation of the task structure, they become less reactive to single feedback events and rely more on integrating past outcomes. This shift is characterized by increased win–stay and fewer lose–shift responses as they increasingly exploit options that maximize gains. In CGT contexts, win–stay can be operationalized as choosing the same deck in two consecutive trials following a win, and lose–shift as switching decks between two consecutive trials following a loss (Garon & English, 2022).

In terms of studies using child-friendly IGT variants, there were only three studies that examined win–stay/lose–shift strategies. Garon and Moore (2007) found that 4-year-olds used adult-like win–stay and lose–shift strategies in their PGT with 30% loss frequency in both decks over 60 trials. They tended to shift more after a loss from the disadvantageous deck compared to the advantageous deck (lose–shift) and stay more after a win from the advantageous deck compared to the disadvantageous deck (win–stay). Later, Garon and English (2022) used a modified PGT with 50% loss frequency in both decks over 50 trials. The 3- to 4-year-olds demonstrated increasing win–stay and decreasing lose–shift behaviors as the game unfolded. For win–stay strategies, 3-year-old children did not differentiate between decks, whereas 4-year-olds stayed more after small wins from the advantageous deck than after large wins from the disadvantageous one. For lose–shift strategies, both age groups shifted more after large losses from the disadvantageous deck than after small losses from the advantageous deck, and this deck-based differentiation became stronger over time, particularly among 4-year-olds. Most comprehensively, Garon and Doucet (2024) identified three distinct profiles of children’s strategy use through latent profile analysis, using the shortened PGT version from the 2022 study with 40 trials. About half of the children (52%, mostly older) exhibited strong deck differentiation, with high win–stay responses for the advantageous deck and high lose–shift responses for the disadvantageous deck, both of which became more pronounced as the game progressed. Approximately one quarter (26%) exhibited no deck differentiation in strategies at all, with an overall high tendency for lose–shift responses. This implies difficulty learning from feedback. The remaining quarter (25%, mostly younger) exhibited maladaptive deck differentiation, paradoxically with high win–stay responses for the disadvantageous deck and high lose–shift responses for the advantageous deck. However, this was moderated by age and task experience. These findings suggest that children become markedly capable of using outcome history to guide their choices during the preschool period.

### 1.3. Individual Differences in Cognitive Self-Regulation: Executive Function and Metacognition

Beyond age-related growth, individual differences in cognitive self-regulation—including executive function (EF) and metacognition—might also influence how children learn from feedback and adjust their strategies across IGT variants. First, EF is a set of top-down, cognitive control processes that involve goal-oriented regulation of thought, action, and emotion (Miyake et al., 2000), which begins to develop in early childhood, with the most rapid increase in the preschool years (Carlson, 2005; Diamond, 2013; Garon, 2016). Hot EF involves emotionally charged, reward-driven situations where impulse control are at stake—like deciding between a small sure reward and a risky larger one. Cool EF, by contrast, operates in more neutral situations and consists of cognitive flexibility (shifting attention between tasks), working memory (holding and updating information), and inhibition (suppressing automatic or prepotent responses) (Zelazo & Carlson, 2012). The IGT has typically been considered a hot EF measure (Toplak et al., 2010), but there is growing literature that performance also depends on cool EF skills (Kouklari et al., 2017; Poland et al., 2016). In particular, in preschool children, the relation between cool EF and performance on IGT variants remains relatively unexplored (Garon, 2016), though some evidence suggests a connection between EF and adaptive decision-making. Hongwanishkul et al. (2005) demonstrated a positive relation between working memory and CGT performance, and Garon et al. (2023) found that cognitive flexibility was associated with performance in the later phase of CGT. Beyond gambling tasks, Kim et al. (2025) showed that children’s cognitive flexibility predicted more adaptive decision-making strategies in a persistence-based choice task.

Second, metacognition—the ability to reflect on and monitor one’s own thinking—represents another dimension of cognitive self-regulation (Roebers, 2017). It consists of declarative knowledge (what you know about how thinking works; Flavell, 1979) and procedural knowledge (Nelson, 1990), which includes monitoring (evaluating how you are performing on a task in real time) and control (regulating your cognitive approach as you go). Garon and colleagues have shown that awareness of the game predicts better performance on PGT (e.g., Garon & Moore, 2007; Garon et al., 2023; Garon & Doucet, 2024). Following Bechara et al. (1997), this awareness can be understood as progressing through phases: “pre-hunch” phase (no explicit knowledge), “hunch” phase (a gut preference without being able to articulate why), and “conceptual” phase (full explicit knowledge), capturing the shift from implicit, affectively guided choices to more deliberate, knowledge-based decisions. In the persistence game by Kim et al. (2025), metacognitive knowledge of the task was linked to strategic performance in addition to EF.

Following Roebers’ (2017) framework, we examined both EF and metacognition as related but distinguishable components of cognitive self-regulation that might each contribute to adaptive decision-making. Stronger EF might help children resist immediate rewards, track outcomes, and shift strategies. Stronger metacognition might help them spot patterns and deliberately guide which strategies to deploy. In this way, cognitive self-regulation might be tied not only to overall better performance but also to more context-appropriate strategy use by flexibly tailoring one’s approach based on feedback and their emerging knowledge of task structure.

### 1.4. Present Study

Despite clear age differences in overall CGT performance, win–stay and lose–shift strategies have not yet been studied in this well-known task. The CGT (Kerr & Zelazo, 2004) and PGT (Garon & English, 2022) are similar—two decks, 50 trials, 50% loss rate, and a conflict between immediate rewards and long-term outcomes—but they differ in ways that might affect how children develop strategies. The CGT results in a net gain of 5 candies for every 10 cards in the advantageous deck and a net loss of 5 candies in the disadvantageous deck (Kerr & Zelazo, 2004), whereas the PGT has more extreme net outcomes, with a net gain of 4 steps (in stairs) per 10 cards in the advantageous deck and a net loss of 10 steps (in stairs) per 10 cards in the disadvantageous deck (Garon & English, 2022). Additionally, the PGT uses a magnetic house visual aid that shows cumulative outcomes continuously, which might reduce working memory demands, whereas the CGT uses edible M&Ms in a graduated cylinder, which might increase motivation through the appeal of food. Given these structural differences, we expected they could elicit somewhat different patterns of strategy use.

Our primary aim was to examine whether 3- to 5-year-olds adjust their win–stay and lose–shift strategies as they learn the CGT’s contingencies. We hypothesized that win–stay would significantly increase over time in the advantageous deck as children learned its long-term value. This prediction is based on Garon and English’s (2022) finding that 4-year-olds increasingly differentiated their win–stay responses by deck, staying more after wins from the advantageous versus disadvantageous deck. However, we hypothesized that win–stay would remain stable over time rather than decrease in the disadvantageous deck. In the first half, children might experience losses and begin to recognize that the deck can yield large penalties, but trials involving no losses (win–no-loss experiences) will be highly attractive, reinforcing them to stay. By the second half, they might have partial awareness that a big loss could occur, but are uncertain, and thus make random choices about whether to stay or not.

For lose–shift strategies, we made two predictions. First, regarding deck differentiation, we hypothesized that lose–shift would be more frequent with the disadvantageous deck than the advantageous deck throughout the game. This aligns with Garon and English’s (2022) finding that both 3- and 4-year-olds showed this deck sensitivity. The more salient losses from the disadvantageous deck (0, 4, 5, or 6 candies) compared to the fewer losses from the advantageous deck (0 or 1 candy) might trigger stronger shifting responses.

Second, regarding temporal change, we hypothesized that lose–shift frequency would remain stable across blocks for both decks. Unlike Garon and English’s (2022) finding that lose–shift declined over time, we did not expect significant changes in our study. This is because, although children can respond to immediate loss differences (supporting deck differentiation), the intermittent loss pattern of the disadvantageous deck (0, 4, 5, or 6 candies) creates inconsistent trial feedback, making it hard for children to develop stable knowledge about which deck is worse. Thus, preschoolers might keep using reflexive lose–shift strategies whenever they hit a loss. Also, because lose–shift is less cognitively demanding than win–stay (Forder & Dyson, 2016; Ivan et al., 2018), this makes it easier for children to maintain lose–shift strategies throughout the task.

Our secondary aim was to determine the degree to which EF and metacognition—as related but distinguishable components of cognitive self-regulation (Roebers, 2017)—would uniquely predict children’s adaptive strategy use in one deck beyond the other. We hypothesized that children with higher EF and/or metacognition would use more win–stay strategies in the advantageous deck (controlling for win–stay in the disadvantageous deck) and more lose–shift strategies in the disadvantageous deck (controlling for lose–shift in the advantageous deck).

## 2. Materials and Methods

### 2.1. Participants

The study included 98 typically developing children aged 3 (*M* = 43.00 months, *SD* = 3.41 months; 14 girls), 4 (*M* = 53.24 months, *SD* = 3.42 months; 23 girls), and 5 years (*M* = 64.85 months, *SD* = 3.86 months; 25 girls), along with their caregivers. All families reside in Minnesota, USA. Participants were recruited through the university’s participant pool, which consists of families who opted in to be contacted about child development research. To be eligible, children had to be proficient in English or bilingual, born full-term, and have no diagnosed developmental disorders (e.g., ADHD or autism). Racial and ethnic composition was white/non-Hispanic (88%) and Asian (1%), with 11% more than one ethnicity. Nearly all children (99%) lived in two- or multiple-caregiver households with married caregivers. Families were largely from high-SES, as indicated by a median household income between $175,000 and $199,999 annually (1 unreported). Most caregivers were highly educated, with 96% of primary caregivers holding a bachelor’s degree or higher.

### 2.2. Procedure

Children and their caregivers attended a single laboratory session at the research university, which lasted about 1 to 1.5 h. Following parental consent and child assent, children played games individually with a female graduate student researcher in a private testing room, where the session was video-recorded. They completed an EF task, a verbal IQ measure, the shortened Children’s Gambling Task (CGT), and the metacognitive interview about the CGT in a fixed order. During this time, caregivers filled out a Family Information Questionnaire on an iPad through Qualtrics. After the session, children received a “junior scientist” T-shirt and a toy, and parents were given a $10 gift card, and parking was complimentary. The study protocol was approved by the university’s Institutional Review Board.

### 2.3. Measures

#### 2.3.1. Children’s Gambling Task (CGT)

**Figure A1:**
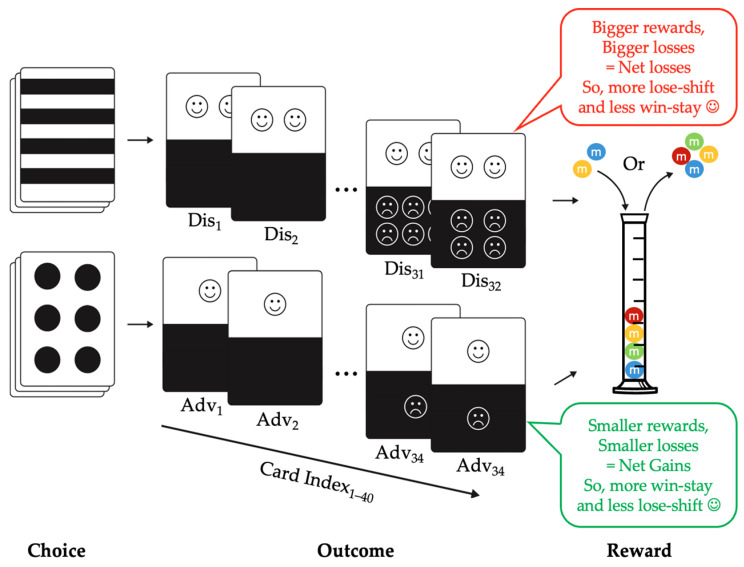
Illustration of Children’s Gambling Task, adapted from Breslav et al. (2022).

A shortened version of the Children’s Gambling Task (CGT; Kerr & Zelazo, 2004) was administered (Appendix A, Figure A1 and Table A1). Children were presented with two decks of laminated cards (each containing 40 cards) positioned side-by-side on a table. A container with mini M&Ms was located nearby, and a transparent graduated cylinder was placed between the decks to display M&M rewards. Each card had its top half visible and its bottom half concealed by a post-it note. The visible portion consistently displayed either one or two happy faces, depending on the deck. Once a child selected a card, the examiner counted the number of happy faces and put an equivalent number of M&Ms into the cylinder. The examiner then removed the post-it note to reveal the number of sad faces and removed an equivalent number of M&Ms from the cylinder. During the demonstration phase, children were shown three cards from each deck, all yielding a net gain of six M&Ms in addition to the first two M&Ms originally put in the cylinder. Following the demonstration, children were asked comprehension questions to confirm their understanding of the game rules—that happy faces represent gains and sad faces represent losses. The one-happy-face deck (advantageous deck) offered one M&M and revealed zero or one sad face, resulting in an average net gain of five M&Ms per block of 10 cards. In contrast, the two-happy-face deck (disadvantageous deck) offered two M&Ms (a larger reward) but revealed zero, four, or six sad faces, resulting in an average net loss of five M&Ms per 10 cards. Although the two-happy-face deck was initially more appealing, continued play revealed its long-term disadvantage. This structure makes the CGT a useful measure of decision-making under uncertainty, as it requires children to adjust their choices based on cumulative feedback. The order of cards and gain/loss contingencies for each deck was identical to those used in the original study by Kerr and Zelazo (2004). The task was shortened from the original 50 trials to 40 based on Kerr and Zelazo’s (2004) observation that preschoolers’ learning patterns were first observed in the third block with trials 30–40, and to reduce testing time and minimize fatigue. All children received the same treat (M&Ms), consistent with Kerr and Zelazo (2004), to standardize the visual appearance of rewards across participants. This ensured that reward increments displayed in the graduated cylinder appeared equivalent for all children.

Following Garon and English (2022), win–stay and lose–shift proportions were calculated (Appendix A Table A2). Strategy variables were determined by examining consecutive trial pairs. Win–stay was operationalized as selecting the same deck on trial n + 1 after winning on trial n (e.g., Advantageous → Advantageous or Disadvantageous → Disadvantageous), and was computed as $\frac{win-stay\;for\;deck}{wins\;for\;deck}$. Lose–shift was operationalized as switching to the alternate deck on trial n + 1 after losing on trial n (e.g., Advantageous → Disadvantageous or Disadvantageous → Advantageous), and was computed as $\frac{lose-shift\;for\;deck}{losses\;for\;deck}$. These proportions were calculated separately for each deck (advantageous vs. disadvantageous) and each block (1st Half: trials 1–20 vs. 2nd Half: 21–40), resulting in eight strategy variables: (1) advantageous deck win–stay in 1st Half block, (2) advantageous deck win–stay in 2nd Half block, (3) disadvantageous deck win–stay in 1st Half block, (4) disadvantageous deck win–stay in 2nd Half block, (5) advantageous deck lose–shift in 1st Half block, (6) advantageous deck lose–shift in 2nd Half block, (7) disadvantageous deck lose–shift in 1st Half block, and (8) disadvantageous deck lose–shift in 2nd Half block. These variables captured both overall strategy use and deck-specific strategic adaptation over time.

#### 2.3.2. Metacognition Interview

Children’s metacognition was measured through an interview format using an awareness test adapted from Garon and Doucet (2024). The questions were slightly modified to fit the context of the CGT. Immediately after completing the CGT, children were asked, (i) “Which card did you think was the best to pick from?”, (ii) “Why do you think it was the best to pick from?”, (iii) “Which card did you think was the worst to pick from?”, and (iv) “Why do you think it was the worst to pick from?” Rather than asking during gameplay as previous studies did (e.g., Garon & Doucet, 2024), we waited until the end. This way, we could capture their declarative form of metacognitive knowledge about the game with full information after experiencing the entire game, without potentially affecting their natural learning process.

Children received a score of 1 for correctly identifying the advantageous or disadvantageous deck, either by pointing or through a verbal response, and a score of 0 if they identified the incorrect deck or expressed uncertainty (e.g., “I don’t know”). Explanations that demonstrated task-relevant reasoning (e.g., for the advantageous deck, noting “fewer sad faces,” “more happy faces”; for the disadvantageous deck, noting “more sad faces” or “fewer happy faces”) were scored as 1. Irrelevant or vague responses (e.g., “I like it,” “I don’t know”, “I think so”) were scored as 0. If a child incorrectly identified the deck, their “why” explanation for that deck was automatically scored as 0. Correct deck identification was a prerequisite for scoring on the explanation. For analysis, the metacognition score was computed by summing the four responses (theoretical range = 0–4).

#### 2.3.3. Minnesota Executive Function Scale (MEFS)

In addition to the metacognition interview, children’s EF skills were assessed. EF was measured using the Minnesota Executive Function Scale (MEFS v.5.4.5; Carlson & Zelazo, 2014), a standardized and normed iPad card-sorting task. The game consists of seven levels that increase in difficulty. The first level is predetermined by the participant’s age. During the task, the examiner demonstrates how to sort cards according to specific rules—for example, dragging cards into boxes that match the card’s color or shape—after which children apply the same rules. Testing ends automatically once a ceiling or basal level is reached. For analysis, age-adjusted standard scores were used (theoretical range = 60–140).

#### 2.3.4. Stanford–Binet Intelligence Scales for Early Childhood (5th)

The verbal knowledge routing subtest of the Stanford–Binet Intelligence Scales for Early Childhood—Fifth Edition (Roid, 2003) was included to estimate children’s verbal intelligence as a potential confound for our analyses. This subtest involves identifying body parts, labeling toys, verbalizing activities depicted in pictures, and defining words. Each question was scored on a 0–1 or 0–2 scale, and the test was terminated after four consecutive errors. For analysis, total raw scores were used (theoretical range = 0–72).

#### 2.3.5. Family Information Questionnaire

Parents completed the Family Information Questionnaire, which gathers demographic details on families, such as household income, living situation (e.g., two-parent household), marital status, education level, and the age, sex, and race/ethnicity of the participating child.

## 3. Results

### 3.1. Preliminary Analyses

All analyses were performed using SPSS 29.0. Table 1 presents correlations and descriptive statistics for the main study variables. Total M&Ms collected served as a validity check, although gameplay win–stay/lose–shift strategies were the primary focus of interest. Table 2 provides detailed descriptive statistics for CGT performance, including win–stay and lose–shift strategy proportions by deck and block, collapsed across age groups and separated by age.

Preliminary analyses examined whether demographic variables should be included as factors in our main analyses with win–stay/lose–shift as dependent variables. One-way ANOVAs examining age group (3-, 4-, 5-year-olds) demonstrated a pattern of effects primarily for lose–shift strategies. Younger children showed more lose–shift strategy use in the 1st Half of trials for both the advantageous deck (*F*(2, 82) = 4.35, *p* = 0.016) and the disadvantageous deck, *F*(2, 82) = 9.05, *p* < 0.001, with marginal effects for the 2nd Half (*p*s = 0.06). No age effects were found for win–stay strategies (all *p*s > 0.16). This pattern—consistent effects on lose–shift but not win–stay, strongest in the 1st Half—suggested that age was meaningfully related to strategy use.

In contrast, independent-samples *t*-tests demonstrated some gender differences, with boys showing higher lose–shift strategies than girls in the 1st Half for the disadvantageous deck, *t*(83) = −1.45, *p* = 0.045, and in the 2nd Half for the advantageous deck, *t*(83) = −2.51, *p* = 0.013. However, effects were limited to these two conditions (all other *p*s > 0.23), indicating an inconsistent pattern. Based on these preliminary findings, age was included in the main analyses, whereas gender was not.

### 3.2. Deck-Specific Strategy Use Across Time

Our first research question examined whether preschoolers would develop deck-specific strategies across time. Specifically, we predicted that (1) win–stay would increase over time in the advantageous deck but remain stable in the disadvantageous deck (Deck × Block interaction), and (2) lose–shift would be higher in the disadvantageous deck compared to the advantageous deck, but would show minimal change across blocks in both decks (Deck main effect but no Block effect, no Deck × Block interaction). To address these questions, we ran two separate mixed ANOVAs—having age group as a between-subject factor and deck and block as within-subject factors—with win–stay proportion and lose–shift proportion as dependent variables in each analysis: 3 (age groups: 3-, 4-, vs. 5-year-olds) × 2 (Decks: Advantageous vs. Disadvantageous) × 2 (Blocks: Trials 1–20 vs. Trials 21–40).

**Figure 1:**
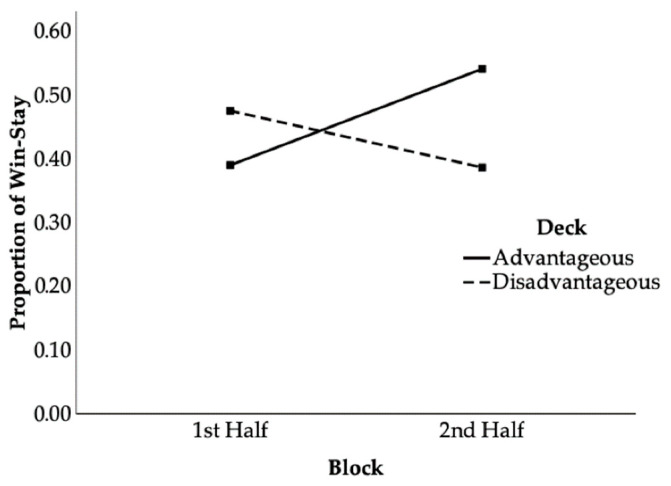
Deck × Block interaction on win–stay strategies.

The ANOVA on win–stay proportion showed no significant main effects of age, *F*(2, 82) = 0.35, *p* = 0.709, *η_p_*^2^ = 0.01, block, *F*(1, 82) = 0.87, *p* = 0.353, *η_p_*^2^ = 0.01, or deck, *F*(1, 82) = 0.38, *p* = 0.538, *η_p_*^2^ = 0.01. Two-way and three-way interactions were also non-significant, except for the Deck × Block interaction: *F*(1, 82) = 17.03, *p* < 0.001, *η_p_*^2^ = 0.17. To follow up this interaction, we conducted multivariate tests to examine simple effects of block within each deck (Figure 1). In the advantageous deck, preschoolers significantly increased win–stay from the 1st Half block (*M* = 0.39, *SE* = 0.04) to the 2nd Half block (*M* = 0.54, *SE* = 0.05): *F*(1, 82) = 13.20, *p* < 0.001, *η_p_*^2^ = 0.14. In the disadvantageous deck, they showed a decrease in win–stay from the 1st Half block (*M* = 0.48, *SE* = 0.04) to the 2nd Half block (*M* = 0.39, *SE* = 0.05), which approached significance: *F*(1, 82) = 3.67, *p* = 0.06, *η_p_*^2^ = 0.04. Supporting our hypothesis, children learned to increasingly exploit the advantageous deck (increased win–stay). Unexpectedly, they also showed a marginal tendency toward reduced perseveration in the disadvantageous deck (decreased win–stay). This learning pattern was evident when pooling all age groups, indicating that even 3-year-olds demonstrated some strategic adaptation.

**Figure 2:**
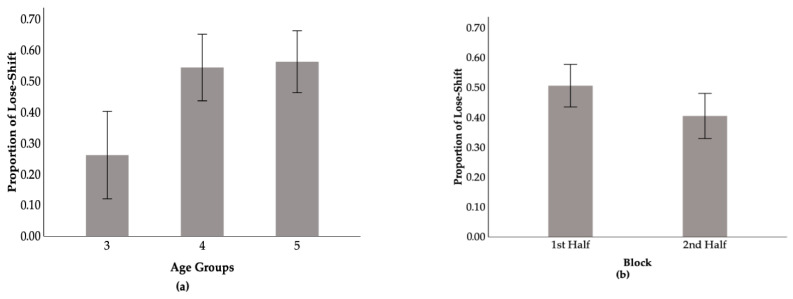
Main effects of (**a**) Age and (**b**) Block on lose–shift strategies. Error bars are 95% confidence intervals.

The ANOVA on lose–shift proportion showed significant main effects of age, *F*(2, 82) = 6.73, *p* = 0.002, *η_p_*^2^ = 0.14, and block, *F*(1, 82) = 12.79, *p* < 0.001, *η_p_*^2^ = 0.14. The main effect of deck was not significant, *F*(1, 82) = 1.44, *p* = 0.233, *η_p_*^2^ = 0.02, nor were any two-way or three-way interactions. Post hoc pairwise comparisons with Bonferroni correction demonstrated that 3-year-olds (*M* = 0.26, *SE* = 0.07) were significantly less likely to lose–shift than both 4-year-olds (*M* = 0.54, *SE* = 0.05; *p* = 0.006) and 5-year-olds (*M* = 0.56, *SE* = 0.06; *p* = 0.003), whereas 4- and 5-year-olds did not differ from each other (Figure 2a). Furthermore, children displayed significantly more lose–shift behavior in the 1st Half block (*M* = 0.51, *SE* = 0.04) compared to the 2nd Half block (*M* = 0.41, *SE* = 0.04) (*p* < 0.001) (Figure 2b). Contrary to our hypothesis, there was no significant deck effect for lose–shift, suggesting that children did not differentiate their lose–shift responses between the advantageous and disadvantageous decks at the group level. Additionally, we did not find the hypothesized stability in lose–shift over time. Rather, lose–shift significantly declined across blocks. Unexpectedly, a developmental pattern was found in that children’s ability to shift away from options after losses improved considerably between ages 3 and 4.

### 3.3. Relations Between Effective Deck-Specific Strategy Use and Cognitive Self-Regulation

Our second research question examined whether EF and metacognition uniquely predicted children’s adaptive, deck-specific strategy use. To address this question, we ran two separate hierarchical linear regression analyses—one predicting advantageous deck win–stay and one predicting disadvantageous deck lose–shift. In each analysis, we entered age and verbal ability in Block 1, strategy use in the opposite deck in Block 2 (i.e., disadvantageous deck win–stay when predicting advantageous deck win–stay; advantageous deck lose–shift when predicting disadvantageous deck lose–shift), and MEFS and metacognition in Block 3. This approach allowed us to test whether EF and metacognition predict adaptive strategy use in a given deck beyond strategy use in the other deck.

For advantageous deck win–stay, metacognition was a unique predictor (*β* = 0.43, *p* = 0.001), whereas EF was not (*β* = −0.07, *p* = 0.579) (Table 3). Age and verbal ability in Block 1 accounted for 1% of variance. Adding disadvantageous deck win–stay in Block 2 accounted for an additional 3% of variance (*ns*). Adding EF and metacognition in Block 3 significantly accounted for an additional 16% of variance. For disadvantageous deck lose–shift, metacognition was again a unique predictor (*β* = 0.34, *p* = 0.002), whereas EF was not (*β* = 0.16, *p* = 0.873) (Table 3). Age and verbal ability in Block 1 accounted for 17% of variance. Adding advantageous deck lose–shift in Block 2 significantly accounted for an additional 20% of variance. Adding EF and metacognition in Block 3 significantly accounted for an additional 11% of variance. These results partially supported our hypothesis: metacognition of task structure—but not general EF—predicted children’s adaptive, deck-specific strategy use.

## 4. Discussion

### 4.1. Increasing Win–Stay Behaviors for Rewarding Options

Supporting our primary hypothesis, children increased their win–stay responses in the advantageous deck across blocks. However, contrary to our hypothesis that win–stay responses would remain stable in the disadvantageous deck, our results indicated a marginally significant decrease across blocks. This suggests children were learning not just to repeat rewarding options but also to avoid perseverating with poorer options. One possible explanation is that children accumulated enough negative experiences with the disadvantageous deck to begin avoiding it even after wins.

These patterns mirror prior findings from the PGT, similar to CGT, where preschoolers gradually increased win–stay responses in advantageous decks and decreased them in disadvantageous decks (e.g., Garon & Doucet, 2024; Garon & English, 2022; Garon & Moore, 2007). It reflects a shift from reactive to more deliberate responding, consistent with the exploration-to-exploitation developmental trajectory (exploration transitioning to exploitation with age; Gopnik, 2020), as repeated positive experiences strengthen action–outcome associations (Sutton & Barto, 2018). However, it is worth noting that the learning was incomplete in that even by the second half, win–stay rates in the advantageous deck were only around 50%.

Interestingly, we did not find significant age differences among 3–5-year-olds in win–stay responses, which was inconsistent with prior studies using the PGT (Garon & Doucet, 2024; Garon & English, 2022). One possible explanation is that the CGT might have held comparable levels of difficulty for all preschoolers. This means that older preschoolers might have more cognitive resources, but the difficult task structure could have restricted performance for all age groups. Compared to other child-friendly gambling tasks like PGT (Garon & Doucet, 2024), the CGT offers only subtle differences in net outcomes between decks (a 10-candy gap per block vs. 14).

### 4.2. Developmental Patterns in Lose–Shift Behaviors

Contrary to our first prediction regarding deck differentiation, children did not show more lose–shift responses in the disadvantageous deck compared to the advantageous deck. This is puzzling, especially given that children differentiated win–stay responses between decks. Our result differs from Garon and English (2022), who found that both 3- and 4-year-olds showed deck sensitivity in lose–shift responses. This discrepancy might be due to loss structure. In CGT, losses are highly variable (0, 4, 5, or 6 candies) in the disadvantageous deck, including trials with no loss at all, which could make deck differences less apparent to young children, compared to the PGT that has more extreme losses (always 6 candies). Moreover, contrary to our second prediction that lose–shift responses would remain constant across blocks regardless of deck, lose–shift actually decreased over time, replicating Garon and English’s (2022) finding. This decline suggests that children became less reactive to individual losses as they gained more experience.

Notably, we observed a developmental pattern that offers insight into how young children process negative feedback: 3-year-olds displayed significantly less lose–shift responses than 4- and 5-year-olds. This contradicts the assumption that young children will shift more after losses, as lose–shift is a simpler, more reflexive strategy (Forder & Dyson, 2016; Ivan et al., 2018). This is possible if 3-year-olds selectively attended to gains more than losses and thus failed to register losses strongly enough to trigger shifting. Research on young children’s judgment suggests that they often show positivity biases in processing information, paying more attention to positive over negative outcomes (Boseovski, 2010). Another possible explanation is that they might lack the cognitive resources to process loss magnitude and maintain knowledge of deck contingencies simultaneously. Certainly, perseveration in the face of continuing losses is a characteristic of pathological gambling (Newman et al., 1987), and there is evidence of similar perseveration among 3-year-old children (Happaney & Zelazo, 2004).

### 4.3. Metacognition Predicts Strategic Learning

Partially supporting our hypothesis, metacognition—but not EF—uniquely predicted deck-specific strategy use, controlling for age, verbal ability, and strategy use in the opposite deck. Children with stronger metacognition used more win–stay strategies in the advantageous deck and more lose–shift strategies in the disadvantageous deck. This suggests that it was not about using more or less of a strategy overall—it was about using strategies appropriately for each context. This finding aligns with theoretical accounts of cognitive self-regulation as involving both EF and metacognition (Roebers, 2017), but suggests these components might play different roles.

We had speculated that EF might help children inhibit the pull of immediate large rewards from the disadvantageous deck, update information in working memory about deck contingencies, and flexibly change their approaches as needed. However, EF did not uniquely predict deck-specific strategy use beyond metacognition. This is somewhat surprising given prior work showing that cool EF correlates with overall gambling task performance (Garon et al., 2023; Hongwanishkul et al., 2005). One possible explanation is that EF might support initial learning of the task structure, but once children develop explicit awareness, metacognition becomes the primary driver of strategic behavior.

### 4.4. Strengths, Limitations and Future Directions

This study has several strengths. First, our study is the first examination of win–stay and lose–shift strategies in the widely used CGT task to our knowledge. Previous studies have examined these strategies using either the PGT with preschoolers (Garon & English, 2022; Garon & Doucet, 2024; Garon & Moore, 2007) or the standard IGT with older populations (Cassotti et al., 2011), but not the CGT. We extended this line of research by analyzing trial-by-trial strategy use in the CGT. Second, our finding that even 3-year-olds demonstrated strategic learning—such as increasing win–stay in the advantageous deck—is encouraging and suggests that its foundation develops much earlier than previously documented. The protracted development of lose–shift, on the other hand, implies that learning from negative outcomes is more challenging in this task. Third, the finding that metacognition—but not EF—predicts effective deck-specific strategy use highlights distinct roles for these components of cognitive self-regulation and identifies metacognition as a key individual difference variable in early adaptive decision-making.

Several limitations should be noted. First, our sample consisted of slightly older preschoolers (M = 4.6 years) who were predominantly female (63%). Future research would benefit from more evenly age-distributed and gender-balanced samples.

Second, children were predominantly white, from two-parent married households, with college-educated and high-income caregivers. This demographic homogeneity limits generalizability and raises several interpretive considerations, so future work should aim for more diverse sample. With respect to reward motivation, it remains unclear whether M&M rewards hold equal motivational value across socioeconomic groups. Research on delay of gratification suggests that children of college-educated mothers wait significantly longer and are more likely to complete delay tasks compared to children of non-college-educated mothers (Watts et al., 2018), possibly due to greater environmental uncertainty among lower-SES children that reduces motivation to wait for future rewards (Kidd et al., 2013). With respect to CGT performance, prior research indicates that Brazilian children from higher-SES backgrounds perform better on later CGT blocks, possibly reflecting stronger ability to integrate feedback over time (Mata et al., 2013b). More recent work found that middle/high-SES Uruguayan children increasingly chose the advantageous deck across blocks, whereas low-SES children did not shift toward advantageous choices even when they had explicit awareness of deck contingencies (Delgado et al., 2022). Nevertheless, this demographic homogeneity carries an interpretative advantage, as the observed age-related and individual differences were more likely due to true developmental or cognitive mechanisms rather than due to underlying sample characteristics, such as SES.

Third, we shortened the CGT from 50 to 40 trials to reduce fatigue, but this might have limited learning opportunities, particularly for younger children who need more experience to extract patterns. Future studies should examine whether increasing the trials would lead to clearer deck differentiation in lose–shift strategies, as well as increased win–stay rates in the advantageous deck (which only reached 50%).

Lastly, we included only one EF measure (MEFS), which leans primarily on cognitive flexibility, and is considered to be a “cool” EF task. It is possible that a more contextualized “hot” EF task would be more strongly related to gambling task performance. In contrast, our metacognition measure concerned the CGT itself. Future research should include a broader battery of EF measures as well as independent measures of declarative and procedural metacognition. We also note that the current binary scoring system, while maximizing clarity and inter-rater reliability, did not capture gradations in the depth or quality of children’s responses. For instance, the current scoring did not distinguish between irrelevant “why” responses and incorrect (but showing partial comprehension) “why” responses, as they both were scored as 0. Future studies should incorporate a more nuanced scoring scheme to capture additional variability in metacognitive sophistication.

## 5. Conclusions

This study examined how preschool-aged children adjust their strategies in response to feedback in an uncertain decision-making context. We specifically investigated preschoolers’ use of win–stay and lose–shift strategies in a shortened version of the CGT, a widely used measure of children’s decision-making. To our knowledge, this is the first study to examine win–stay and lose–shift strategies in the CGT. We found that children increasingly stayed with the advantageous deck after wins but decreased lose–shift strategies over time. Overall, 3-year-olds showed substantially more lose–shift strategies than 4- and 5-year-olds. Controlling for age, verbal ability, and strategy use in the opposite deck, metacognition—but not EF—predicted children’s ability to tailor strategies to the deck environment. This suggests that knowing what works might matter more than general cognitive control for tailoring strategies to the task at hand. Future research should extend these findings with more diverse samples and broader measures of both EF and metacognition.

## Figures and Tables

**Table 1:** Bivariate correlations and descriptive statistics of main study variables.

	Age	Verbal IQ	MEFS	Metacognition	Total M&Ms
Verbal IQ	0.61 **	–			
MEFS	−0.08	0.20	–		
Metacognition	0.25 *	0.16	0.17	–	
Total M&Ms	0.12	−0.06	0.03	0.48 **	–
Range	36–71	0–28	90–119	0–4	−21–24
*M*	55.47	19.44	102.59	1.60	0.49
*SD*	9.45	4.56	5.95	1.52	10.95

Age in months. MEFS = Minnesota Executive Function Scale standard score. Regarding total M&Ms, the minimum value in the range is negative, reflecting cumulative losses that exceeded prior gains; in practice, the cylinder was empty at these points. * *p* < 0.05 ** *p* < 0.01.

**Table 2:** Detailed descriptive statistics for CGT performance.

Age Groups	Block	Win–Stay		Lose–Shift	
		Adv *M* (*SD*)	Dis *M* (*SD*)	Adv *M* (*SD*)	Dis *M* (*SD*)
All	1st	0.41(0.34)	0.46 (0.38)	0.53 (0.39)	0.57 (0.39)
	2nd	0.55 (0.40)	0.36 (0.41)	0.41 (0.39)	0.46 (0.40)
3	1st	0.29 (0.41)	0.54 (0.45)	0.29 (0.43)	0.26 (0.36)
	2nd	0.50 (0.46)	0.52 (0.49)	0.23 (0.38)	0.26 (0.37)
4	1st	0.43 (0.34)	0.49 (0.39)	0.57 (0.38)	0.63 (0.37)
	2nd	0.59 (0.38)	0.32 (0.36)	0.49 (0.39)	0.49 (0.37)
5	1st	0.45 (0.31)	0.40 (0.32)	0.61 (0.36)	0.68 (0.34)
	2nd	0.53 (0.40)	0.31 (0.40)	0.44 (0.37)	0.52 (0.41)

Adv = advantageous deck; Dis = disadvantageous deck; 1st = 1st Half; 2nd = 2nd Half; Adv Win–stay = stay after a win from the advantageous deck; Adv Lose–shift = shift after a loss from the advantageous deck; Dis Win–stay = stay after a win from the disadvantageous deck; Dis Lose–shift = shift after a loss from the disadvantageous deck.

**Table 3:** Hierarchical linear regression results predicting effective deck-specific strategies.

Predictors		Advantageous Deck Win–Stay						Disadvantageous Deck Lose–Shift				
	*B* (*SE*)	β	*t*	*p*	*R* ^2^	∆ *R* ^2^	*B* (*SE*)	β	*t*	*P*	*R* ^2^	∆ *R* ^2^
Block 1					0.01	–					0.17 **	–
Age	0.00 (0.00)	0.07	0.46	0.648			0.02 (0.01)	0.48	3.23	0.002		
Verbal IQ	−0.01 (0.01)	−0.08	−0.51	0.613			−0.01 (0.01)	−0.15	−0.98	0.331		
Block 2					0.04	0.03					0.36	0.20 **
Age	0.00 (0.01)	0.12	0.73	0.468			0.01 (0.01)	0.38	2.85	0.006		
Verbal IQ	−0.01 (0.01)	−0.11	−0.66	0.511			−0.01 (0.01)	−0.16	−1.24	0.218		
Opp Deck	0.18 (0.13)	0.18	1.41	0.165			0.46 (0.11)	0.46	4.29	<0.001		
Block 3					0.20	0.16 **					0.47	0.11 **
Age	0.00 (0.01)	0.00	0.02	0.981			0.01 (0.01)	0.29	2.22	0.030		
Verbal IQ	−0.01 (0.01)	−0.09	−0.57	0.569			−0.01 (0.01)	−0.17	−1.37	0.178		
Opp Deck	0.22 (0.12)	0.23	1.90	0.062			0.50 (0.10)	0.50	4.97	<0.001		
MEFS	−0.00 (0.01)	−0.07	−0.56	0.579			0.00 (0.01)	0.02	0.16	0.873		
Metacognition	0.10 (0.03)	0.43	3.41	0.001			0.08 (0.02)	0.34	3.33	0.002		

Age = age in months. MEFS = Minnesota Executive Function Scale standard score. Opp Deck = Opposite deck strategy use; refers to the disadvantageous deck win–stay for the Advantageous Win–Stay model, and the advantageous deck lose–shift for the Disadvantageous Lose–Shift model. ** *p* < 0.01.

## Data Availability

The raw data supporting the conclusions of this article will be made available by the authors on request.

## Abbreviations

The following abbreviations are used in this manuscript:

CGT	Children’s Gambling Task
EF	Executive Function
IGT	Iowa Gambling Task
MEFS	Minnesota Executive Function Scale
PGT	Preschool Gambling Task
SES	Socioeconomic Status

## Appendix A

**Table A1:** Fixed loss schedule for Children’s Gambling Task, reproduced from Kerr and Zelazo (2004). No. = card number; Dis = disadvantageous deck; Adv = advantageous deck.

No.	Dis	Adv	No.	Dis	Adv	No.	Dis	Adv	No.	Dis	Adv
1	0	0	11	0	0	21	0	0	31	−6	0
2	0	0	12	−6	−1	22	−6	0	32	−4	0
3	−4	−1	13	0	−1	23	0	0	33	−5	0
4	0	0	14	−5	0	24	−6	−1	34	0	−1
5	−6	−1	15	−4	0	25	0	−1	35	0	−1
6	0	0	16	0	0	26	−4	−1	36	0	0
7	−4	−1	17	−6	−1	27	−5	0	37	−4	−1
8	0	0	18	−4	−1	28	−4	0	38	−6	0
9	−5	−1	19	0	0	29	0	−1	39	0	−1
10	−6	−1	20	0	−1	30	0	−1	40	0	−1

**Table A2:** An example of how to compute win–stay and lose–shift scores for each deck and each block, adapted from Garon and Doucet (2024). A = advantageous; D = disadvantageous; W = win feedback (gains > losses); L = loss feedback (gains < losses); WS = win–stay; LS = lose–shift. An illustration of coding the strategy variable for Trials 1 and 2 is as follows, colored in blue step-by-step: Step 1. On a row where a child’s deck choice is recorded for each trial, locate Trials 1 and 2, and find which choice a child made on Trial 1. It was an advantageous deck. Step 2. Check whether it was a win or a loss. It was a win. Step 3. Check which choice a child made on Trial 2. It was the advantageous deck. Step 4. Locate a row with trial pairs and code 1 for A–WS’ Trial Pairs 1 and 2 (i.e., the child chose the advantageous deck, received a win feedback, and then decided to stay).

## References

[B1-behavsci-16-00023] Aïte A., Cassotti M., Rossi S., Poirel N., Lubin A., Houdé O., Moutier S. (2012). Is human decision making under ambiguity guided by loss frequency regardless of the costs? A developmental study using the soochow gambling task. Journal of Experimental Child Psychology.

[B2-behavsci-16-00023] Andrews G., Moussaumai J. (2015). Improving children’s affective decision making in the children’s gambling task. Journal of Experimental Child Psychology.

[B3-behavsci-16-00023] Bechara A., Damasio A. R., Damasio H., Anderson S. (1994). Insensitivity to future consequences following damage to human prefrontal cortex. Cognition.

[B4-behavsci-16-00023] Bechara A., Damasio H., Tranel D., Damasio A. R. (1997). Deciding advantageously before knowing the advantageous strategy. Science.

[B5-behavsci-16-00023] Boseovski J. J. (2010). Evidence for “Rose-colored glasses”: An examination of the positivity bias in young children’s personality judgments. Child Development Perspectives.

[B6-behavsci-16-00023] Breslav A. D. S., Zucker N. L., Schechter J. C., Majors A., Bidopia T., Fuemmeler B. F., Kollins S. H., Huettel S. A. (2022). Shuffle the decks: Children are sensitive to incidental nonrandom structure in a sequential-choice task. Psychological Science.

[B7-behavsci-16-00023] Bunch K. M., Andrews G. (2012). Development of relational processing in hot and cool tasks. Developmental Neuropsychology.

[B8-behavsci-16-00023] Bunch K. M., Andrews G., Halford G. S. (2007). Complexity effects on the children’s gambling task. Cognitive Development.

[B9-behavsci-16-00023] Carlson S. M. (2005). Developmentally sensitive measures of executive function in preschool children. Developmental Neuropsychology.

[B10-behavsci-16-00023] Carlson S. M., Zelazo P. D. (2014). Minnesota executive function scale: Test manual.

[B11-behavsci-16-00023] Casey B. J., Somerville L. H., Gotlib I. H., Ayduk O., Franklin N. T., Askren M. K., Jonides J., Berman M. G., Wilson N. L., Teslovich T., Glover G., Zayas V., Mischel W., Shoda Y. (2011). Behavioral and neural correlates of delay of gratification 40 years later. Proceedings of the National Academy of Sciences.

[B12-behavsci-16-00023] Cassotti M., Houdé O., Moutier S. (2011). Developmental changes of win-stay and loss-shift strategies in decision making. Child Neuropsychology.

[B13-behavsci-16-00023] Crone E. A., van der Molen M. W. (2004). Developmental changes in real life decision making: Performance on a gambling task previously shown to depend on the ventromedial prefrontal cortex. Developmental Neuropsychology.

[B14-behavsci-16-00023] Daw N. D., O’Doherty J. P., Dayan P., Seymour B., Dolan R. J. (2006). Cortical substrates for exploratory decisions in humans. Nature.

[B15-behavsci-16-00023] Defoe I. N., Dubas J. S., Figner B., van Aken M. A. G. (2015). A meta-analysis on age differences in risky decision making: Adolescents versus children and adults. Psychological Bulletin.

[B16-behavsci-16-00023] Delgado H., Aldecosea C., Menéndez Ñ., Rodríguez R., Nin V., Lipina S., Carboni A. (2022). Socioeconomic status differences in children’s affective decision-making: The role of awareness in the children’s gambling task. Developmental Psychology.

[B17-behavsci-16-00023] Diamond A. (2013). Executive functions. Annual Review of Psychology.

[B18-behavsci-16-00023] Flavell J. H. (1979). Metacognition and cognitive monitoring: A new area of cognitive–developmental inquiry. American Psychologist.

[B19-behavsci-16-00023] Forder L., Dyson B. J. (2016). Behavioural and neural modulation of win-stay but not lose-shift strategies as a function of outcome value in rock, paper, scissors. Scientific Reports.

[B20-behavsci-16-00023] Gao S., Wei Y., Bai J., Lin C., Li H. (2009). Young children’s affective decision-making in a gambling task: Does difficulty in learning the gain/loss schedule matter?. Cognitive Development.

[B21-behavsci-16-00023] Garon N. (2016). A review of hot executive functions in preschoolers. Journal of Self-Regulation and Regulation.

[B22-behavsci-16-00023] Garon N., Doucet E. (2024). To explore or exploit: Individual differences in preschool decision making. Cognitive Development.

[B23-behavsci-16-00023] Garon N., English S. D. (2022). Heterogeneity of decision-making strategies for preschoolers on a variant of the IGT. Applied Neuropsychology: Child.

[B24-behavsci-16-00023] Garon N., Hecker O., Kwan A., Crocker T. A., English S. D. (2023). Integrated versus trial specific focus improves decision-making in older preschoolers. Child Neuropsychology.

[B25-behavsci-16-00023] Garon N., Moore C. (2004). Complex decision-making in early childhood. Brain and Cognition.

[B26-behavsci-16-00023] Garon N., Moore C. (2007). Developmental and gender differences in future-oriented decision-making during the preschool period. Child Neuropsychology.

[B27-behavsci-16-00023] Gopnik A. (2020). Childhood as a solution to explore–exploit tensions. Philosophical Transactions of the Royal Society B: Biological Sciences.

[B28-behavsci-16-00023] Happaney K., Zelazo P. D. (2004). Resistance to extinction: A measure of orbitofrontal function suitable for children?. Brain and Cognition.

[B29-behavsci-16-00023] Heilman R. M., Miu A. C., Benga O. (2008). Developmental and sex-related differences in preschoolers’ affective decision making. Child Neuropsychology.

[B30-behavsci-16-00023] Hongwanishkul D., Happaney K. R., Lee W. S. C., Zelazo P. D. (2005). Assessment of hot and cool executive function in young children: Age-related changes and individual differences. Developmental Neuropsychology.

[B31-behavsci-16-00023] Ivan V. E., Banks P. J., Goodfellow K., Gruber A. J. (2018). Lose-shift responding in humans is promoted by increased cognitive load. Frontiers in Integrative Neuroscience.

[B32-behavsci-16-00023] Kerr A., Zelazo P. D. (2004). Development of “hot” executive function: The children’s gambling task. Brain and Cognition.

[B33-behavsci-16-00023] Kidd C., Palmeri H., Aslin R. N. (2013). Rational snacking: Young children’s decision-making on the marshmallow task is moderated by beliefs about environmental reliability. Cognition.

[B34-behavsci-16-00023] Kim S., Berry D., Carlson S. M. (2025). Should I stay or should I go? Children’s persistence in the context of diminishing rewards. Developmental Science.

[B35-behavsci-16-00023] Kim S., Carlson S. M. (2024). Understanding explore-exploit dynamics in child development: Current insights and future directions. Frontiers in Developmental Psychology.

[B36-behavsci-16-00023] Kouklari E.-C., Thompson T., Monks C. P., Tsermentseli S. (2017). Hot and cool executive function and its relation to theory of mind in children with and without autism spectrum disorder. Journal of Cognition and Development.

[B37-behavsci-16-00023] Mata F., Sallum I., de Moraes P. H. P., Miranda D. M., Malloy-Diniz L. F. (2013a). Development of a computerised version of the children’s gambling task for the evaluation of affective decision-making in Brazilian preschool children. Estudos de Psicologia (Natal).

[B38-behavsci-16-00023] Mata F., Sallum I., Miranda D. M., Bechara A., Malloy-Diniz L. F. (2013b). Do general intellectual functioning and socioeconomic status account for performance on the children’s gambling task?. Frontiers in Neuroscience.

[B39-behavsci-16-00023] Mischel W., Berkowitz L. (1974). Processes in delay of gratification. Advances in experimental social psychology.

[B40-behavsci-16-00023] Miyake A., Friedman N. P., Emerson M. J., Witzki A. H., Howerter A., Wager T. D. (2000). The unity and diversity of executive functions and their contributions to complex “Frontal Lobe” tasks: A latent variable analysis. Cognitive Psychology.

[B41-behavsci-16-00023] Nelson T. O. (1990). Metamemory: A theoretical framework and new findings. Psychology of learning and motivation.

[B42-behavsci-16-00023] Newman J. P., Patterson C. M., Kosson D. S. (1987). Response perseveration in psychopaths. Journal of Abnormal Psychology.

[B43-behavsci-16-00023] Nussenbaum K., Hartley C. A. (2019). Reinforcement learning across development: What insights can we draw from a decade of research?. Developmental Cognitive Neuroscience.

[B44-behavsci-16-00023] Overman W. H. (2004). Sex differences in early childhood, adolescence, and adulthood on cognitive tasks that rely on orbital prefrontal cortex. Brain and Cognition.

[B45-behavsci-16-00023] Paulus M. P., Hozack N., Zauscher B. E., McDowell J. E., Frank L. D., Brown G. M., Braff D. L. (2001). Prefrontal, parietal, and temporal cortex networks underlie decision-making in the presence of uncertainty. NeuroImage.

[B46-behavsci-16-00023] Poland S. E., Monks C. P., Tsermentseli S. (2016). Cool and hot executive function as predictors of aggression in early childhood: Differentiating between the function and form of aggression. British Journal of Developmental Psychology.

[B47-behavsci-16-00023] Prencipe A., Zelazo P. D. (2005). Development of affective decision making for self and other: Evidence for the integration of first- and third-person perspectives. Psychological Science.

[B48-behavsci-16-00023] Roebers C. M. (2017). Executive function and metacognition: Towards a unifying framework of cognitive self-regulation. Developmental Review.

[B49-behavsci-16-00023] Roid G. H. (2003). Stanford-Binet intelligence scales for early childhood.

[B50-behavsci-16-00023] Sutton R. S., Barto A. (2018). Reinforcement learning: An introduction.

[B51-behavsci-16-00023] Thompson C., Barresi J., Moore C. (1997). The development of future-oriented prudence and altruism in preschoolers. Cognitive Development.

[B52-behavsci-16-00023] Toplak M. E., Sorge G. B., Benoit A., West R. F., Stanovich K. E. (2010). Decision-making and cognitive abilities: A review of associations between Iowa Gambling Task performance, executive functions, and intelligence. Clinical Psychology Review.

[B53-behavsci-16-00023] Watts T. W., Duncan G. J., Quan H. (2018). Revisiting the marshmallow test: A conceptual replication investigating links between early delay of gratification and later outcomes. Psychological Science.

[B54-behavsci-16-00023] Wurm F., Walentowska W., Ernst B., Severo M. C., Pourtois G., Steinhauser M. (2022). Task learnability modulates surprise but not valence processing for reinforcement learning in probabilistic choice tasks. Journal of Cognitive Neuroscience.

[B55-behavsci-16-00023] Zelazo P. D., Carlson S. M. (2012). Hot and cool executive function in childhood and adolescence: Development and plasticity. Child Development Perspectives.

